# Exposure of *Anopheles* mosquitoes to trypanosomes reduces reproductive fitness and enhances susceptibility to *Plasmodium*

**DOI:** 10.1371/journal.pntd.0008059

**Published:** 2020-02-07

**Authors:** Constentin Dieme, Natalia Marta Zmarlak, Emma Brito-Fravallo, Christelle Travaillé, Adrien Pain, Floriane Cherrier, Corinne Genève, Estefanía Calvo-Alvarez, Michelle M. Riehle, Kenneth D. Vernick, Brice Rotureau, Christian Mitri

**Affiliations:** 1 Genetics and Genomics of Insect Vectors Unit, Department of Parasites and Insect Vectors, Institut Pasteur, Paris, France; 2 Centre National de la Recherche Scientifique, UMR2000, Paris, France; 3 Trypanosome Transmission Group, Trypanosome Cell Biology Unit, Department of Parasites and Insect Vectors, INSERM U1201, Institut Pasteur, Paris, France; 4 Graduate School of Life Sciences ED515, Sorbonne Universities, UPMC Paris VI, Paris, France; 5 Institut Pasteur–Bioinformatics and Biostatistics Hub–C3BI, USR 3756 IP CNRS–Paris, France; 6 Department of Microbiology and Immunology, Medical College of Wisconsin, Milwaukee, Wisconsin, United States of America; National Institute of Allergy and Infectious Diseases, UNITED STATES

## Abstract

During a blood meal, female *Anopheles* mosquitoes are potentially exposed to diverse microbes in addition to the malaria parasite, *Plasmodium*. Human and animal African trypanosomiases are frequently co-endemic with malaria in Africa. It is not known whether exposure of *Anopheles* to trypanosomes influences their fitness or ability to transmit *Plasmodium*. Using cell and molecular biology approaches, we found that *Trypanosoma brucei brucei* parasites survive for at least 48h after infectious blood meal in the midgut of the major malaria vector, *Anopheles coluzzii* before being cleared. This transient survival of trypanosomes in the midgut is correlated with a dysbiosis, an alteration in the abundance of the enteric bacterial flora in *Anopheles coluzzii*. Using a developmental biology approach, we found that the presence of live trypanosomes in mosquito midguts also reduces their reproductive fitness, as it impairs the viability of laid eggs by affecting their hatching. Furthermore, we found that *Anopheles* exposure to trypanosomes enhances their vector competence for *Plasmodium*, as it increases their infection prevalence. A transcriptomic analysis revealed that expression of only two *Anopheles* immune genes are modulated during trypanosome exposure and that the increased susceptibility to *Plasmodium* was microbiome-dependent, while the reproductive fitness cost was dependent only on the presence of live trypanosomes but was microbiome independent. Taken together, these results demonstrate multiple effects upon *Anopheles* vector competence for *Plasmodium* caused by eukaryotic microbes interacting with the host and its microbiome, which may in turn have implications for malaria control strategies in co-endemic areas.

## Author summary

In nature, females *Anopheles* mosquitoes that transmit the malaria parasites *Plasmodium*, take successive blood meals to maximize their offspring. During these blood meals, mosquitoes are exposed to a variety of microbes present in the host blood in addition to *Plasmodium*, the obligate parasite that causes malaria. The *Trypanosoma* parasites, causing trypanosomiases, are sympatric with the malaria parasites in numerous African regions, therefore, a single female mosquito could be in contact with both pathogens concurrently or through successive blood meals. In this work, we showed that exposure of females *Anopheles* mosquitoes to *Trypanosoma* enhanced their susceptibility to malaria parasites, reduced their reproductive fitness and modulated their bacterial gut flora. While the effect of trypanosomes ingestion on *Plasmodium* infection is microbiome dependent, the phenotype on the reproductive fitness is microbiome independent. These results highlight the need for considering the effect of eukaryotic microbes on *Anopheles* biology for malaria control strategies.

## Introduction

Vector borne diseases such as malaria, trypanosomiases, and others are often sympatric in Africa [1]. Therefore, in these areas, successive blood meals taken by adult *Anopheles* female mosquitoes during their life could increase their exposure to different microorganisms, including parasites, bacteria and viruses [1].

In areas with the highest malaria burden, such as in Sub-Saharan Africa, *Plasmodium falciparum* transmission depends on the complex ecological determinants (biotic and abiotic factors) that drive population dynamics of the primary African vectors *Anopheles gambiae s*.*l*. and *A*. *funestus* but also mosquito intrinsic factors for pathogen development [2–7]. Eukaryotic microbes, including microsporidia, gregarines and trypanosomatids, have been identified in mosquitoes. However, relatively little attention has been paid to *Anopheles* vector interactions with eukaryotic microbes and their potential impact on *Plasmodium* development in the mosquito [3].

African trypanosomiases (Human and Animal African Trypanosomiases) are neglected tropical diseases caused by *Trypanosoma* species most exclusively transmitted to humans and their livestock by the bite of both male and female tsetse flies of the genus *Glossina*. These diseases have devastating socio-economic consequences for Sub-Saharan Africa with 13 million people and about 50 million cattle at risk [8–10]. Between 1990 and 2015, almost 440,000 Human African Trypanosomiases (HAT) cases were reported [11]. At the same time, sustained international control efforts have reduced the number of new HAT cases to only 2,163 in 2016 [12]. Nevertheless, probably more cases remain undetected given that sleeping sickness tends to occur in remote rural areas and that latent infections in humans or animals could represent a significant proportion of the infected populations that would act as reservoirs for the parasites [13] [14].

Although *Trypanosoma* and *Plasmodium* infections have been extensively examined separately in their respective vectors, little is known about the possible effects of *Anopheles* mosquito co-exposures to both parasite species, successively or concurrently. Patients in endemic countries can be co-infected with *Plasmodium* and *Trypanosoma* parasites [15]. Therefore, concomitant exposures to *Plasmodium* and *Trypanosoma* in *Anopheles* vectors are theoretically possible in Africa. Moreover, the epidemiological roles of latent human cases and animal reservoirs in HAT endemic foci [9,13,16] are not well-documented and the current burden of Animal African Trypanosomiases (AAT) is a concern. In addition, recent studies on *A*. *gambiae* feeding behavior have shown high proportions of (i) blood meals actually taken on a single non-human host and (ii) mixed blood meals taken from both animals and humans [17,18]. Therefore, successive exposures of *Anopheles* vectors to *Trypanosoma* ingested from animals and *Plasmodium* ingested from infected humans are likely to occur. Based on all of this information, we hypothesized that in co-endemic areas where *Anopheles* mosquitoes are exposed to *Trypanosoma brucei brucei* in bloodmeals from animal hosts, these parasites could modulate the micro-environment of the mosquito midgut, potentially influencing the development of *Plasmodium* in the midgut [1].

Here, using *Anopheles coluzzii*, a member of the *A*. *gambiae* complex, we show that *T*. *b*. *brucei* bloodstream forms survive for at least 48h post-feeding in the *A*. *coluzzii* midgut before being cleared. This survival was sufficient to impact the abundance of the bacterial gut flora as well as mosquito reproductive fitness. In addition, *T*. *b*. *brucei* ingestion significantly increased *Plasmodium* infection prevalence in a microbiota-dependent manner. These findings suggest that *T*. *b*. *brucei* parasites affect a potential interplay between the gut microbiota and reproduction of *A*. *coluzzii* and, more importantly, that it could directly increase the risk of malaria parasite transmission.

## Methods

### Ethic statement

This study was conducted in strict accordance with the recommendations from the Guide for the Care and Use of Laboratory Animals of the European Union (European Directive 2010/63/UE) and the French Government. The protocol was approved by the “Comité d’éthique en expérimentation animale de l’Institut Pasteur” CETEA 89 (Permit number: 2013–0129), by the French Ministry of Scientific Research (Permit number: 202195.02) and undertaken in compliance with Institut Pasteur Biosafety Committee (protocol CHSCT 14.114).

### Mosquitoes

*A*. *coluzzii* colony Fd03 initiated in Mali [19] was reared at 26°C and 80% humidity, on a 12 h light/dark cycle with access to cotton soaked in 10% sucrose solution, in insectaries of the Unit of Genetics and Genomics of Insect Vectors of the Institut Pasteur Paris, France.

### Mouse infections with *Trypanosoma brucei brucei*

All experiments were performed with three-week-old female Swiss *Mus musculus* mice (Janvier, France). *T*. *b*. *brucei* AnTat 1.1E fluorescent bloodstream forms expressing a cytosolic chimeric reporter including a red fluorescent marker and a red-shifted bioluminescent marker (PpyREH9/TY1/TdTomato) were cultivated in HMI9 medium supplemented with 10% fetal calf serum at 37°C in 5% CO_2_ [20]. Parasitemia was assayed by automated fluorescent cell counting with a Muse cytometer (Merck-Millipore, detection limit 5.10^2^ parasites/ml) according to the manufacturer’s recommendations. Parasites were counted, centrifuged and resuspended at 10^7^cells/ml. Then, 10^6^ parasites of this suspension were inoculated by intra-peritoneal (IP) injection to each mouse. Mice parasitemia was assessed under an inverted light microscope Leica DMIL (Leica) with standardized single-use hemocytometers (Hycor Kova, detection limit 10^4^ parasites/ml) according to the manufacturer’s recommendations or by automated fluorescent cell counting with a Muse cytometer (Merck-Millipore).

### Mouse infections with *Plasmodium yoelii*

Mice were inoculated with 10^5^ red blood cells (RBCs) infected with GFP-transgenic *P*. *yoelii* strain, GFP@HSP70-GOMO [21]. Four days post-injection, blood samples were taken from the tail, and parasitemia was determined by flow cytometry. Furthermore, male gametocyte maturity was verified by performing an exflagellation test as previously described [22].

### *T*. *b*. *brucei* survival in mosquito midgut

To assess *T*. *b*. *brucei* survival in mosquito midgut, mosquitoes were allowed to feed on cultured fluorescent *T*. *b*. *brucei* AnTat1.1E bloodstream forms. 119 and 68 mosquitoes were dissected at 2 and 5 days post-feeding, respectively, and entire midguts were examined by fluorescence microscopy to detect living red fluorescent parasites. Dissected midguts were scored twice for the presence of trypanosomes either 2 or 5 days post-feeding on mice.

### Mosquito infection with the rodent malaria parasite *P*. *yoelii*

For all experiments, mice infected with *P*. *yoelii*, strain GFP@HSP70-GOMO at 5–6% parasitemia with mature gametocytes, were used. Mice were first anaesthetized by intraperitoneal (IP) injection of ketamine (Imalgene 1000 at 125 mg/kg) and xylazine (Rompun 2% at 12.5 mg/kg) before mosquitoes were allowed to feed for 30 min. Unfed mosquitoes were discarded and fed mosquitoes were maintained at 24°C (*P*. *yoelii*) and 70% relative humidity on 10% sucrose solution as previously described [23]. 3 independent biological experiments were performed.

### Concomitant *A*. *coluzzii* co-infections with *T*. *b*. *brucei* and *P*. *yoelii*

Mice were infected either with *P*. *yoelii* or co-infected with both *P*. *yoelii* and *T*. *b*. *brucei*. For co-infected mice, *T*. *b*. *brucei* were inoculated 1 day after *P*. *yoelii* injection. 4 days post *P*. *yoelii* infections in mice, 2 groups of *A*. *coluzzii* mosquitoes were fed either on a *P*. *yoelii* mono-infected mouse that served as control or on a co-infected mouse. All mosquitoes that were not visibly engorged were removed. Mosquito midguts were dissected and infection status was checked by fluorescence microscopy looking at the oocyst stage 8 days post-feeding.

### Successive *A*. *coluzzii* infections with *T*. *b*. *brucei* and *P*. *yoelii* with and without antibiotics

*A*. *coluzzii* females were allowed to feed either on mice mono-infected with *T*. *b*. *brucei* or on naive mice (as negative control). Five days later, all mosquitoes were given a second blood meal on mice infected with *P*. *yoelii*. For each feeding, all mosquitoes that were not visibly engorged were removed. Samples were dissected at 8 days after *P*. *yoelii* challenge to confirm infection status at the oocyst stage. Throughout the experiment mosquitoes were maintained under antibiotic pressure as previously described [24]. Briefly, immediately following adult emergence, mosquitoes were maintained on a 10% sucrose solution complemented with Penicillin 62.5 μg/mL, Streptomycin 100 μg/mL and gentamicin 50 μg/mL, and this solution was changed every day. Successive co-infections were performed as described above.

### Successive *A*. *coluzzii* co-infections with *T*. *b*. *brucei* and *P*. *falciparum*

Gametocytes from cultured *P*. *falciparum* isolate NF54 were produced by the CEPIA mosquito infection facility of the Institut Pasteur, as described previously [23]. *A*. *coluzzii* females were allowed to feed either on mice mono-infected with *T*. *b*. *brucei* or on naive mice (as negative control). Five days after feeding, all mosquitoes were given a second blood meal on cultured *P*. *falciparum* gametocytes, as described previously [25]. Unfed females were discarded and only fully engorged females were maintained at 26°C and at 70% relative humidity on 10% sucrose solution supplemented with 0.05% para-amino benzoic acid.

### Assessing the direct effect of *T*. *b*. *brucei* on the gut microbiome and the reproductive fitness with cultured parasites

The pleomorphic *T*.*b*. *brucei* AnTat1.1E bloodstream forms were cultured in HMI9 medium as described above. In order to obtain stumpy forms, the parasite stage infective to the insect host, an *in vitro* induction was performed in slender forms cultured at 1.10^5^ parasites/mL with the cAMP analogue 8-pCPT-2′-O-Me-cAMP (Biolog, Germany) at 5μM for 48h [26]. The presence of stumpy forms was verified by using the anti-PAD1 rabbit polyclonal antibody (1:250, Keith Matthews, Edinburgh, UK) targeting the carboxylate-transporter Proteins Associated with Differentiation 1 (PAD1) [27]. Differentiated parasites were centrifuged and resuspended in commercial mechanically defibrinated sheep blood (BCL, France) at 10^8^ parasites/mL in order to feed *Anophele*s females through a membrane feeding system in which the blood mixture is maintained at 37°C. Mosquito guts were collected 48h and 5 days post-trypanosome ingestion. These experiments were performed to remove any potential confounding factors from the blood of infected mice and be able to attribute results to the direct effect of trypanosomes on the mosquitoes.

### *Plasmodium* infection phenotype

Midguts were dissected 8 days after infection. For *P*. *falciparum*, midguts were stained in 0.4% mercurochrome and the number of oocysts was counted under a contrast light microscope (Nikon Eclipse Ni). For GFP-fluorescent *P*. *yoelii* (strain GFP@HSP70-GOMO), midgut oocysts were directly counted under a fluorescent binocular stereo microscope (Nikon SMZ18), using a GFP filter (450-500nm for absorbance spectra; 510 nm for emission). Prevalence of infection is defined as the proportion of infected mosquitoes among the total number of dissected mosquitoes. Infection intensity is defined as the number of oocysts per mosquito, determined using only those mosquitoes harbouring at least one oocyst. Uninfected mosquitoes were excluded from the analysis of infection intensity.

### Quantitative polymerase chain reaction (qPCR)

Using cDNA or genomic DNA, all qPCRs were performed as described in [25], using SYBR green supermix (KAPA SYBR FAST ABI, from Sigma-Aldrich) and the CFX96 Touch Real-Time PCR Detection System (from Biorad). Ribosomal protein *AgrpS7* gene (F_5’-CACCGCCGTGTACGATGCCA-3’ and R_5’-ATGGTGGTCTGCTGGTTCTT-3’) was used as an internal control and the spliced-leader (SL) RNA (F_5’-CAATATAGTACAGAAACTG-3’ and R_ 5’-AACTAACGCTATTATTAGAA-3’) was used to confirm the infection status of each sample by *T*. *b*. *brucei*. The quantification of each gene was obtained as a ratio of the *AgrpS7*. Analysis of the expression of transcript relative to *rpS7* was performed according to the 2−ΔΔCt method [28]. PCR condition run are: 95°C for 5min, then 40 cycle of (95°C for 15 sec, 60°C for 1min (plateread)), 60°C for 30 sec.

### Microbiota analysis by qPCR

Dissection was performed as described previously [29]. Briefly, before dissection, mosquitoes were washed in 75% ethanol for 5 min, and washed three times in sterile PBS to wash out non-attached bacteria, thus preventing sample contamination with cuticle bacteria during dissection. For each biological replicate, 20 midguts were collected from each mosquito group (-Tryp) and (+Tryp), frozen immediately on dry ice and stored at −80°C until processing. To assess antibiotic effectiveness, 20 mosquito midguts from each group (-Tryp) and (+Tryp) were collected 48h post-feeding. Midguts were excluded from the analysis when they appeared to have burst, resulting in a substantial loss of the gut content. DNA was extracted with DNeasy PowerSoil Kit (QIAGEN). The V4 region of the 16S rDNA (16S_V4q_F: 5’-GTGCCAGCMGCCGCGGTAA-3’ and 16S_V4q_R: 5’-GGACTACHVGGGTWTCTAAT -3’) was used for measuring the total bacterial abundance by quantitative PCR. For Enterobacteriaceae family detection, we used two different primer pairs: 16SrDNA [30] (Entero_16S_F: 5’ CGTCGCAAGMMCAAAGAG 3’- and Entero_16S_R: 5’TTACCGCGGCTGCTGGCAC3’) and 23SrDNA (-Entero_23S_F: 5’-TGCCGTAACTTCGGGAGAAGGCA-3’ and Entero_23S_R: 5’ -TCAAGGACCAGTGTTCAGTGTC- 3’) [31,32]. DNA samples from each independent biological replicate were used to perform distinct qPCR in triplicate and fold changes obtained between (-Tryp) and (+Tryp) were combined as a mean and illustrated graphically.

### Effect of trypanosome ingestion on the reproductive fitness

#### *A*. *coluzzii vitellogenin* expression with and without antibiotics

Mosquitoes were collected at day 2 post feeding on *Trypanosoma*-infected or naive mice. For mosquitoes treated with antibiotics, the protocol described above was used. Total RNA was extracted with TRIzol reagent (Invitrogen) from pools of 10 mosquitoes from each batch. cDNAs were generated using M-MLV reverse transcriptase (Invitrogen) from total RNAs. qPCR was performed to quantify differences in *vitellogenin* gene expression between *A*. *coluzzii* mosquitoes fed on *Trypanosoma-*infected mice and naive mice or between mosquitoes co-infected with *T*. *b*. *brucei/P*. *yoelii* and those infected only with *P*. *yoelii*. The primers Vg_qF: 5’-CCGACTACGACCAGGACTTCC-3’ and Vg_qR: 5’ CACTGGACGACACGTACGGGC-3’ were used as target for qPCR.

#### Effect of trypanosome ingestion on fecundity and egg hatching

Egg laying was assessed for both individual females and for pools of 20 females exposed (+Tryp) or not exposed (-Tryp) to *T*. *brucei brucei*. For the pools of females, the number of laid eggs was counted 72h post-blood feeding and divided by the number of females (20 gravid females) to get an average number of eggs laid per female, as a proportion. The differences in these proportions between -Tryp and +Tryp groups were tested using Chi-Squared tests in 3 independent experiments. For the eggs laid by individual females that were placed in individual pot with a single male, the number of eggs was counted individually and differences between the two groups were tested using a non-parametric Wilcoxon signed-rank non-parametric test.

To measure the egg hatching rate without bias, we have randomly collected eggs freshly laid by about 10 individual female “mothers” (<5 eggs per individual female) of both the (-Tryp) and the (+Tryp) groups, put these eggs in multiplate wells containing tap water, stored them at 26°C, and monitored their hatching daily. The numbers of hatched eggs (into larvae) were counted from day 2 to day 4 post-egg laying. The number of hatched eggs from each group (-Tryp or +Tryp) was counted and divided by the number of initial eggs put in water, as a proportion. Differences in these proportions between (-Tryp) and (+Tryp) groups were tested using a Chi-Squared test.

### RNA sequencing and analysis

Two batches of *Anopheles* mosquitoes have been fed on mice, non-infected (control) or mono-infected with red fluorescent *T*. *b*. *brucei*. For each condition, a pool of 10 infected-mosquitoes was collected at 24h and 48h post-feeding. Total RNA extractions from intact mosquitoes were performed on each pool using TRIzol reagent (Invitrogen), yielding a total of 12 samples. All RNA-seq was performed at the University of Minnesota Genomics Center (genomics.umn.edu).

### Details for RNA sequencing experiment and analysis

Three independent biological replicates were performed, and for each experimental replicate, two batches of *Anopheles* mosquitoes have been fed on mice, non-infected (naive feeding, control) or mono-infected with red fluorescent *T*. *b*. *brucei* (*Trypanosoma*-infectious feeding). For each condition, a pool of 10 infected-mosquitoes was collected at 24h and 48h post-feeding. Total RNA extractions from intact mosquitoes were performed on each pool, yielding 12 samples (see Table 1 above).

**Table 1:** Experimental plan for RNA sequencing. Two groups of mosquitoes were fed for one on naive mouse (uninfected control) and for the other on trypanosome-infected mouse. 10 mosquitoes were collected at 24h and 48h post-feeding and used for total RNA extraction. The experiment as reproduced three times (“Experiment 1”, “Experiment 2” and “Experiment 3”).

	Naive feeding		*Trypanosoma* infectious feeding	
	24h post-feeding	48h post-feeding	24h post-feeding	48h post-feeding
**Experiment 1**	Naive mouse 1	Naive mouse 1	Trypa_mouse_1	Trypa_mouse_1
**Experiment 2**	Naive mouse 2	Naive mouse 2	Trypa_mouse_2	Trypa_mouse_2
**Experiment 3**	Naive mouse 3	Naive mouse 3	Trypa_mouse_3	Trypa_mouse_3

Briefly, using Illumina’s Truseq RNA Sample Preparation Kit (Cat. # RS-122-2001), 1 microgram of total RNA was oligo-dT purified using oligo-dT coated magnetic beads, fragmented and then reverse transcribed into cDNA. The cDNA was fragmented, blunt-ended, and ligated to indexed (barcoded) adaptors and amplified using 15 cycles of PCR. Final library size distribution was validated using capillary electrophoresis and quantified using fluorimetry (PicoGreen) and via q-PCR. Indexed libraries were then normalized, pooled and then size selected to 320bp +/- 5% using Caliper’s XT instrument. Truseq libraries were hybridized to a paired end flow cell and individual fragments were clonally amplified by bridge amplification on the Illumina cBot. Once clustering was complete, the flow cell was loaded on the HiSeq 2000 and sequenced using Illumina’s SBS chemistry. Primary analysis of sequence reads and demultiplexing were done using CASAVA 1.8.2 and de-multiplexed FASTQ files were used for downstream analyses.

The quality of the raw reads was checked with FastQC version 0.11.5 (http://www.bioinformatics.babraham.ac.uk/projects/fastqc) and multiqc version 0.7 [33] BWA-mem version 0.7.7-r441 (https://arxiv.org/abs/1303.3997) with default parameters was used for alignment against the reference genome of *Anopheles gambiae* str. PEST version AgamP4 (from VectorBase). Genes were counted using featureCounts [34] version 1.4.6-p3 with the annotation version AgamP4.7 and the parameters–t mRNA–g ID.

Counts data were analyzed using R version 3.3.1 (R Core Team. R: A Language and Environment for Statistical Computing, R Foundation for Statistical Computing (2016)) and the Bioconductor package DESeq2 version 1.14.1 [35] using default parameters. A generalized linear model including time (24h and 48h), treatment (non-infected, *T. b*. *brucei* infected), hatching effect and the interaction term between time and treatment was applied in order to test for (i) inter-condition differences and (ii) the time-treatment interaction. For each pairwise comparison, raw p-values were adjusted for multiple testing using the Benjamini and Hochberg procedure [36]. Genes with adjusted p-values below 0.05 were considered differentially expressed. Sequence data related to analysis have been submitted in ArrayExpress under this link:

https://www.ebi.ac.uk/arrayexpress/experiments/E-MTAB-7469

ArrayExpress accession: E-MTAB-7469

Username: adrien.pain@pasteur.fr

Password: xwceYPkk

### Immunofluorescence assays

Trypanosomes were isolated from mosquito midguts in phosphate buffered saline (PBS) 1X and placed on poly-L-lysine coated slides and fixed in methanol at -20°C for 5 seconds before being re-hydrated in PBS for 10 minutes as previously described [37]. *Trypanosoma* parasites were co-stained in PBS containing 0.1% bovine serum albumin for 45 minutes at 37°C with the two following antibodies: (1) the anti-CRD rabbit polyclonal antibody (1:300) targeting the cross-reactive determinant of the glycosylphosphatidylinositol anchor of surface membrane proteins, predominantly the variant surface glycoproteins of trypanosome bloodstream forms [38], and (2) the anti-EP mouse IgG1 monoclonal antibody (1:500) (CLP001A, Cedarlane, Canada) targeting the procyclin surface coat of procyclic trypomastigotes. Species-specific secondary antibodies coupled to AlexaFluor594 or 488 (Jackson ImmunoResearch, USA) were then used in PBS containing 0.1% bovine serum albumin for 30 minutes at 37°C. DNA was stained with 4,6-diamidino-2-phenylindole (DAPI) and slides were mounted under cover slips with ProLong antifade reagent (Invitrogen) as previously described [37]. Image acquisition was carried out under a Leica DMI 4000B automated inverted epifluorescence microscope (LEICA, Germany) with a 100x objective and equipped for bright field and phase contrast imaging, using a Prime95B CMOS camera (PhotoMetrix) controlled by Micro-manager 1.4 (NIH). Image intensities were standardized (image displays were set to the same minimum and maximum intensities in order to homogenize their presentation and allow visual comparisons) and analyzed with ImageJ 1.49 (NIH).

### Statistical analyses

Differences in infection prevalence and egg hatching rates were statistically tested using Chi-Square tests. For differences in oocyst load (infection intensity) and in the number of eggs per individual females, we used Wilcoxon signed-rank non-parametric tests. Statistical differences in prevalence and intensity were first tested independently for each independent replicate as described above, and p-values were empirically determined using 10^5^ Monte-Carlo permutations. Following independent statistical tests, the p-values from independent tests of significance were combined using the meta-analytical approach of Fisher [39] when the direction of change for each independent replicate was concordant (e.g., each independent replicate displayed higher infection prevalence than their paired -Tryp controls). Statistical analyses were done using R [40]. For qPCR analysis of the expression of transcripts relative to rpS7, the 2−ΔΔCt method was used. Difference in deltaCt distribution across the independent biological replicates between (-Tryp) and (+Tryp) samples was statistically tested using Student t-test.

## Results

### *T*. *b*. *brucei* differentiates into procyclic forms and survives at least 48h in *A*. *coluzzii* midgut

To investigate the survival of *T*. *b*. *brucei* bloodstream forms in mosquitoes, *A*. *coluzzii* mosquitoes were allowed to feed on cultured fluorescent *T*. *b*. *brucei* bloodstream forms. Mosquitoes were dissected at day 2 and 5 post-feeding. Results revealed that ingested *T*. *b*. *brucei* parasites survived at least 48h in *A*. *coluzzii* midgut for 81.5% of fed females (Table 2). Interestingly, fluorescent midguts were also observed in 2.9% of the mosquitoes five days after trypanosome ingestion (Table 2).

**Table 2:** *T*. *b*. *brucei* bloodstream forms survived in *A*. *coluzzii* midguts at least 48h post-ingestion. Mosquito midguts were extracted at day 2 and day 5 post-feeding. Results of three independent experiments are shown in the table, where the numbers (No.) of positive midguts (carrying fluorescent *T*. *b*. *brucei* parasites) over the total number of dissected mosquitoes were counted. The prevalence column shows the proportion (in %) of mosquito midguts carrying fluorescent trypanosomes, at day 2 and day 5 post-trypanosomes feeding. The mean of prevalence is shown in bold for both day 2 and day 5 time points. The lower total numbers of mosquitoes at day 5 are not due to increased mosquito mortality but to the sampling design.

	Day 2 post-feeding		Day 5 post-feeding	
	No. positive midguts / No. total midguts	Prevalence (%)	No. positive midguts / No. total midguts	Prevalence (%)
**Experiment 1**	26/28	92.8	1/24	4.2
**Experiment 2**	49/60	81.7	1/14	7.1
**Experiment 3**	22/31	71.0	0/30	0.0
**Total / Mean**	97/119	**81.5**	2/68	**2.9**

**Fig 1:**
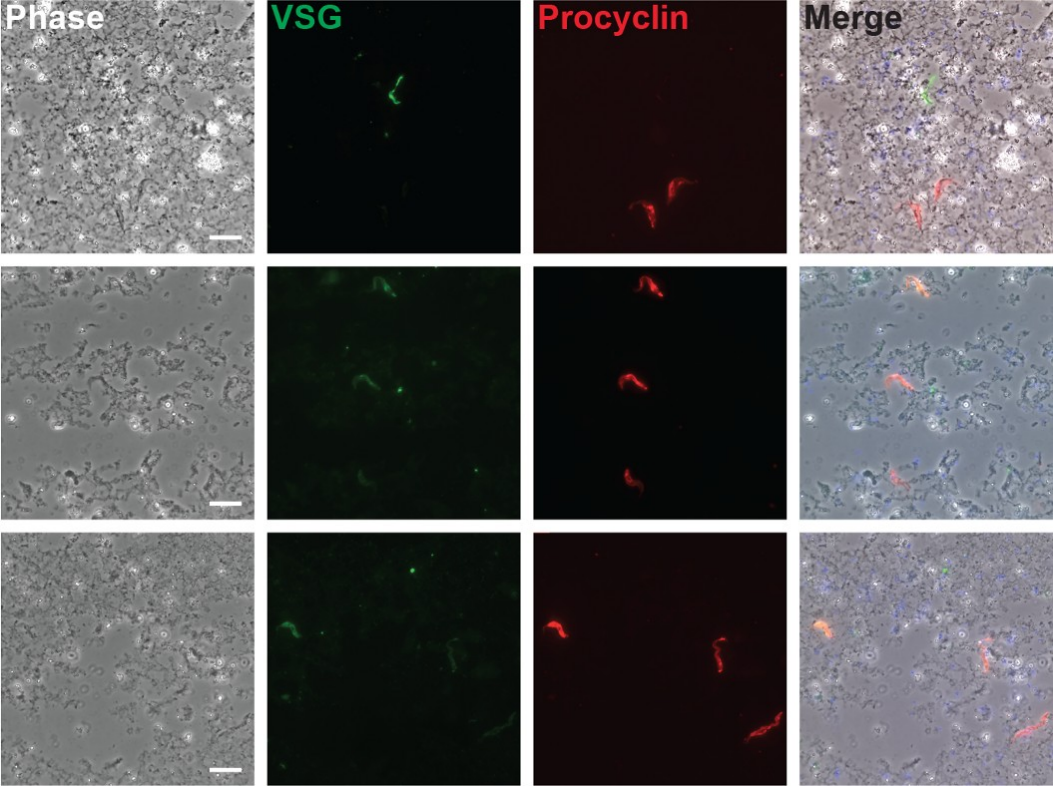
*T*. *b*. *brucei* bloodstream forms differentiate into procyclic forms within 24h in the *Anopheles* midgut. A double immunofluorescence assay was performed using an antibody specifically targeting the bloodstream form VSG’s cross-reactive determinant (anti-CRD in green) and an anti-procyclin antibody labelling the procyclin surface coat (anti-EP in red) on trypanosomes extracted from mosquito midguts at 24h post-ingestion. Each image line illustrates a distinct microscopic field. Whereas only a few parasites were positive for VSGs alone, a strong procyclin signal was observed for a majority of parasites. Some parasites were positive for both antibodies, suggesting an ongoing surface coat switching. Scale bars indicate 10 micrometers.

Knowing this, we assessed whether wild-type bloodstream stumpy trypanosomes can differentiate into procyclic forms in the mosquitoes. A double immunofluorescence assay was performed on trypanosomes extracted from mosquito midguts at 24h post-ingestion using an antibody specifically targeting the bloodstream Variant Surface Glycoprotein (VSG) coat (anti-CRD) [38] and an anti-procyclin antibody labelling the procyclic surface coat [41] on trypanosomes extracted from mosquito midguts at 24h post-ingestion. While only few parasites were presenting a positive signal for VSGs, we observed a strong procyclin signal on a majority of parasites from midguts collected at 24h (Fig 1). We also performed videos of mosquito midguts dissected 48h post-trypanosome ingestion (S1 Movie) and, in all midguts where the red fluorescence was detected, trypanosomes with the morphology and size of procyclic trypomastigote were observed actively swimming. These results indicate that trypanosomes readily differentiate from stumpy to procyclic stages during the first 24h in the mosquito midgut.

We hypothesized that a microorganism viable for at least 48h and with such motility in the mosquito midgut might significantly affect the midgut micro-environment and possibly impact i) the gut bacterial flora, ii) the mosquito immune response and/or iii) the mosquito fitness. Therefore, we successively tested these three hypotheses.

### *Trypanosoma* ingestion increases the mosquito gut microbiota abundance

**Fig 2:**
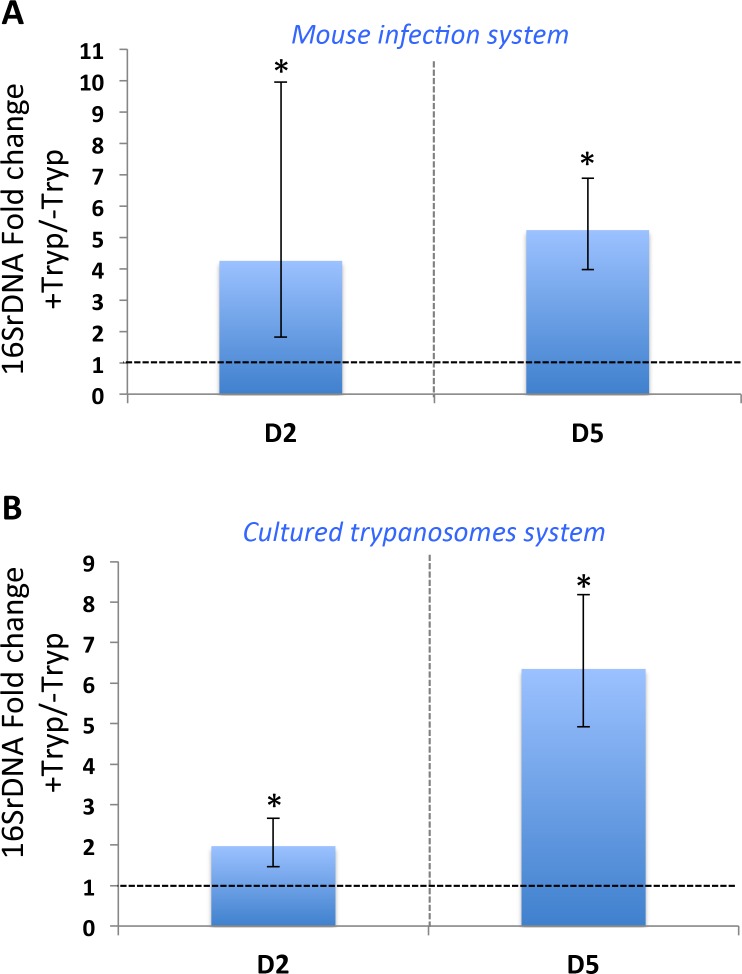
*Trypanosoma* ingestion increases *A*. *coluzzii* bacterial flora abundance at day 2 and day 5 post-feeding. The graphs in A and B show median fold change of the total bacteria load in the mosquito midgut using 16S rDNA detection by qPCR and the ribosomal protein *rps7* gene as the internal calibrator. qPCR detection was performed at day 2 (D2) and day 5 (D5) post-blood meal. The graph in A shows the effect on the mosquito microbiome from females fed on the mouse infection system. The graph in B shows the effect on the mosquito microbiome from females fed on cultured trypanosomes mixed with sheep blood. -Tryp = mosquitoes fed on blood without trypanosomes; +Tryp = mosquitoes fed on blood containing trypanosomes. The dotted line represents the level of 16S rDNA from -Tryp mosquitoes. The ratio of the normalized 16S rDNA detection in “+Tryp” *versus* “-Tryp” was calculated using triplicates from the same cDNA dilution. Error bars show median absolute deviation computed by permutation from 3 experiments. *: statistically significant p-value (p<0.05) related to the deltaCt distribution between “+Tryp” and “-Tryp”.

We monitored the bacterial load in the mosquito midgut at day 2 and day 5 post-feeding on *Trypanosoma-*infected mice or naive mice (control group). Quantitative PCR (qPCR) targeting the 16S ribosomal RNA gene (16S rDNA) revealed a large proliferation of bacteria in mosquitoes exposed to *T*. *b*. *brucei*, with a 5-fold increase of the total enteric bacterial population abundance at day 2 and day 5 after the blood meal (Fig 2A). Similar increases in the microbiome at both times points were observed when mosquitoes were allowed to feed on cultured trypanosomes added to commercial sheep blood as compared to sheep blood without trypanosomes (Fig 2B). This indicates that the effect we observed on the microbiome when using the mouse infection system (Fig 2A) was not the result of potential mice factors, but instead is due to a direct effect of trypanosome parasites.

### *Trypanosoma* ingestion weakly modulates *Anopheles* immune factors

To query the effects of *T*. *b*. *brucei* survival in the mosquito midgut, RNA sequencing was used to identify host transcriptional response to *Trypanosoma* ingestion at 24h and 48h post-trypanosome ingestion. While no effect was observed in the transcriptional response to trypanosome ingestion at 24h (S2 Table), only 13 genes were significantly differentially expressed at 48 h after a trypanosome-containing bloodmeal as compared to naive bloodmeal (S1 Table). Most of the candidate genes do not have an annotated or predicted function related to immunity, except for two genes: the leucine-rich repeat protein *APL1B* (AGAP007035) and the 3-glucan binding protein *GNBPB1* (AGAP004455) (S1 Table).

Measurement by RT-qPCR in independent RNA samples confirmed that only *APL1B* expression was increased by two-fold after trypanosome ingestion (S1A Fig), however this expression increase was not statistically significant between (-Tryp) and (+ Tryp) groups. Nevertheless, although this was not statistically significant, the weak induction of *APL1B* expression after trypanosome exposure was abolished when mosquitoes were treated with antibiotics (S1B Fig), indicating that *APL1B* expression responds to augmented bacterial abundance rather than to the presence of trypanosomes. Thus, ingestion of trypanosomes modulated very weakly and indirectly the expression of one immune factor through an influence on the enteric flora.

### *Trypanosoma* ingestion affects the reproductive fitness in *A*. *coluzzii*

**Fig 3:**
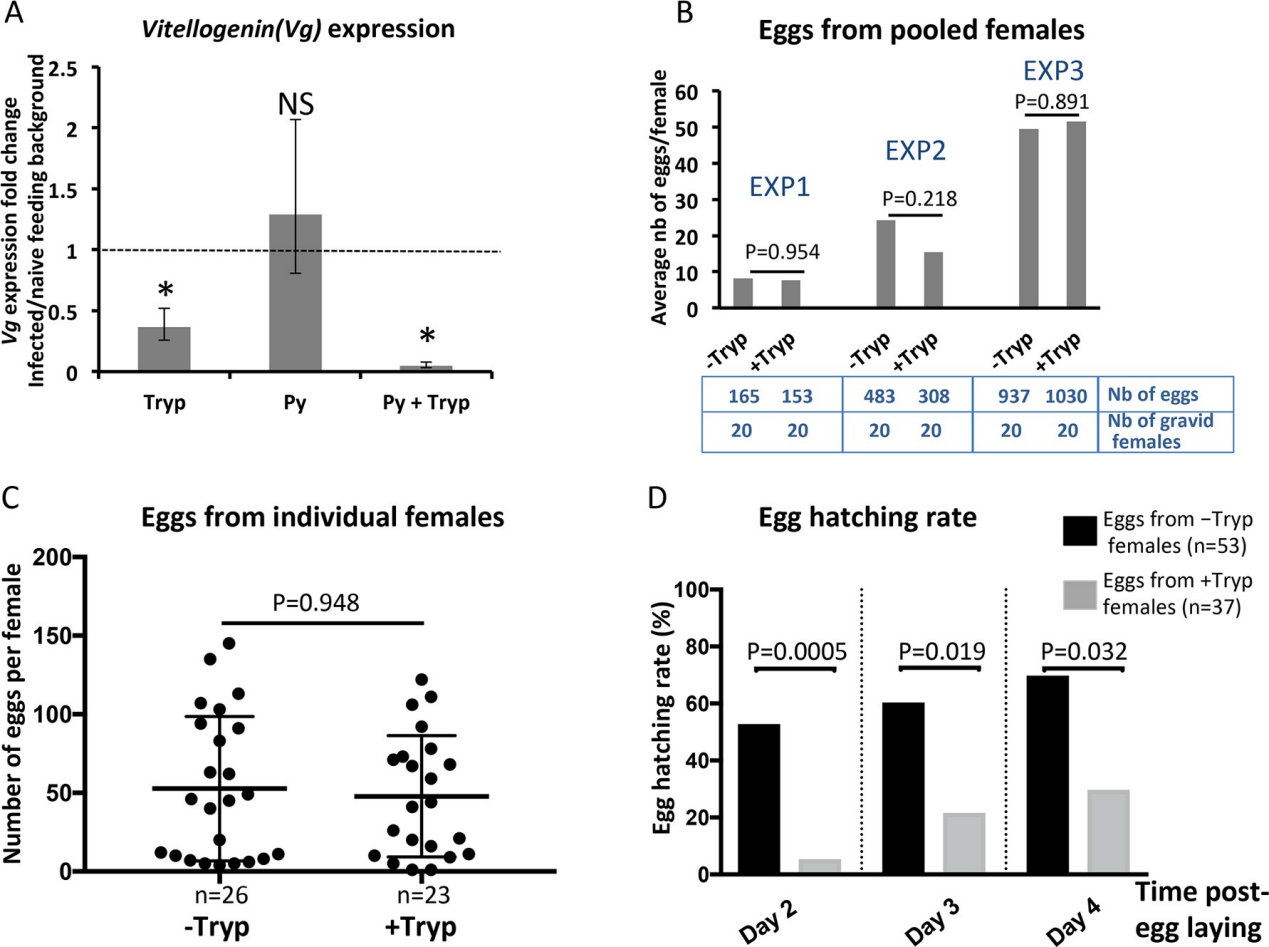
*Trypanosoma* ingestion reduces the reproductive fitness in *A*. *coluzzii*. (A) The graph shows median fold change of *vitellogenin* expression in mosquitoes fed on mice infected by *Trypanosoma* (Tryp) or by *P*. *yoelii* (Py) or by both *Trypanosoma* and *P*. *yoelii* (Py+Tryp) as compared to those fed on naive mice (doted line). The ribosomal protein *rps7* gene was used as an internal calibrator. *Vitellogenin* expression decreased in mosquitoes that fed on mice mono-infected by *T*. *b*. *brucei* (Tryp) and in those that fed on mice co-infected by both *T*. *b*. *brucei* and *P*. *yoelii* (Py+Tryp), as compared to those fed on a naive mouse. The ratio of the normalized *vitellogenin* expression in Tryp, Py, Py+Tryp *versus* naive control was computed using triplicates from the same cDNA dilution. Error bars show median absolute deviation computed by permutation from 3 independent replicates. *: Statistically significant p-value (p<0.05) related to the deltaCt distribution between “+Tryp” and “-Tryp”. NS: Non-significant p-value. (B) The graph shows the average number of eggs per female using pools of females exposed (+Tryp) or not (-Tryp) to *T*. *b. brucei* in three independent replicates. The table below the graph shows, for each experiment, the number of counted eggs and the number of gravid females exposed (+Tryp) or not (-Tryp) to trypanosome parasites. The p-values obtained from a Chi-square test show that there is no statistical significant effect on egg laying. (C) A comparable, yet refined experiment, was performed with the number of eggs counted from each individual gravid female and the difference between the two groups of females was analysed using a Wilcoxon signed-rank non-parametric tests; n = number of individual females from each group. (D) The graph shows the hatching rate of eggs collected from females exposed (grey bar) or not exposed (black bar) to trypanosome parasites at Day 2 to Day 4 post-egg laying. The p-values obtained from a Chi-squared test show that there is a statistical significant impact on the hatching rates between exposed (grey bar) *versus* non-exposed (black bar) females; n = number of eggs from each group that were placed in individual wells containing water.

Mosquito egg maturation induces the expression of the gene encoding for the precursor of the major yolk protein, *vitellogenin* (*Vg*) through the activation of the 20-hydroxyecdysone (20E) pathway [42–44]. In order to measure the effect of trypanosome ingestion on mosquito reproductive fitness, we measured expression of *Vg* 48h after feeding on blood with or without trypanosomes. *Vg* expression decreased significantly in mosquitoes exposed to trypanosomes but was not modified by another eukaryotic microorganism, such as *P*. *yoelii*, when it was ingested alone (Fig 3A). To strengthen this finding, we also tested the expression of another lipid transporter, the Lipophorin (Lp), another yolk protein precursor in mosquitoes [45,46], and also controlled via the 20E pathway [44]. We observed that, as for *Vg* expression, *Lp* expression is decreased 48h post trypanosome ingestion (S2A Fig).

Then we collected eggs from both pooled and individual *A*. *coluzzii* females fed on either trypanosome-infected (+Tryp) or naive mice (-Tryp), and we observed that trypanosome ingestion did not affect *Anopheles* fecundity, defined as the number of laid eggs per females, for either pooled females (Fig 3B) or individual ones (Fig 3C).

Despite no difference in the number of eggs laid, due to the fact that trypanosome ingestion significantly affects *Vg* and *Lp* expression (Fig 3A), we also compared the egg-hatching rate between the two mosquito populations, which represents the capacity of laid eggs to develop into larvae, i.e. the female fertility. Eggs from each female group (-Tryp and +Tryp) were collected at 72h post-blood feeding and placed individually in well containing tap water. The number of viable larvae was counted from day 2 to day 4 post-egg laying. The egg-hatching rate was significantly lower in eggs from +Tryp females as compared to eggs from -Tryp females (Fig 3D). This indicates that, while trypanosome ingestion does not affect female fecundity (i.e. the number of eggs laid), it affects the viability of the laid eggs (i.e. female fertility) by altering their capacity to develop into larvae. This is likely due to a functional failure related to the observed decrease of both *Vg* and *Lp* expression.

In order to remove any potential confounding factors from the blood of infected mice and be able to attribute results to the direct effect of trypanosomes on the mosquito reproductive fitness, cultured *T*. *b*. *brucei* parasites were mixed with commercial sheep blood (for control, commercial sheep blood without trypanosomes was used) and used to feed mosquitoes. We first assessed *Vg* and *Lp* expressions at 48h, and we observed a strong decrease in expression of both genes in the +Tryp group as compared to the -Tryp group (S2B Fig). Then, we collected eggs from individual females to assess their fecundity (number of laid eggs/female) and similarly to the mouse infection system, we did not observe any difference between +Tryp and -Tryp groups in the number of laid eggs (S2C Fig). These results indicate that the effects on both *Vg* and *Lp* decreased expressions, and more widely on the mosquito reproductive fitness, are neither due to potential immune or metabolic factors nor to anaemia from trypanosome-infected mice.

### *Trypanosoma* ingestion increases *A*. *coluzzii* susceptibility to *P*. *yoelii* and *P*. *falciparum*

As *Trypanosoma* ingestion strongly affects the abundance of the enteric microbiome and the expression of *Vg* in *A*. *coluzzii*, which have both been described to modulate *Anopheles* competence for *Plasmodium* [47–49], we hypothesized that trypanosome parasites could also impact the development of *Plasmodium* during either subsequent or concomitant exposure.

**Fig 4:**
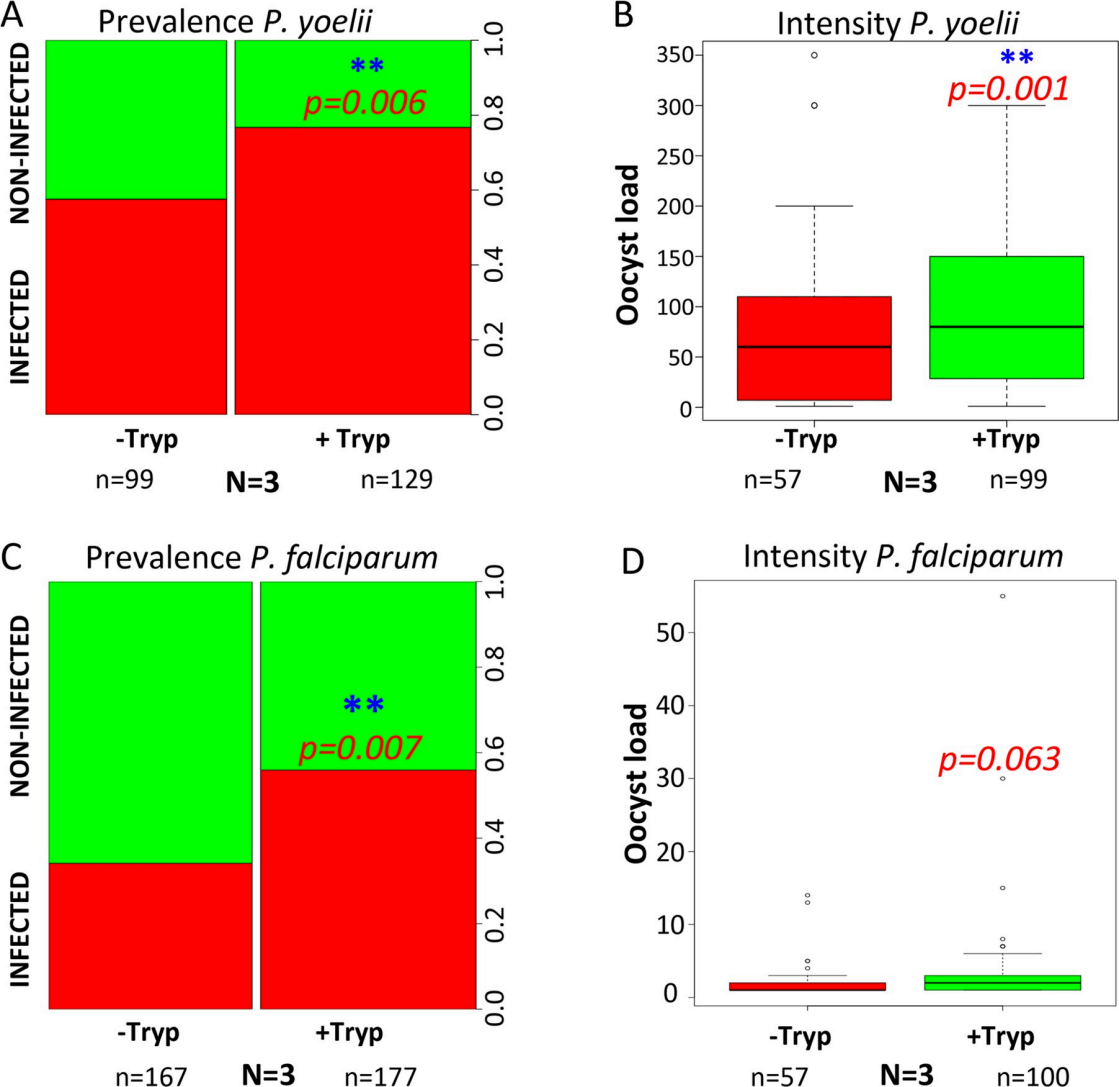
A first infectious blood meal with *T*. *b*. *brucei* increases mosquito susceptibility to rodent and human malaria parasites. Panels (A) & (B) show results of infection prevalence and infection intensity for *P*. *yoelii*, respectively. Panels (C) & (D) show results of infection prevalence and infection intensity for *P*. *falciparum*, respectively. -Tryp = group of mosquitoes previously fed on a naive mouse (without *Trypanosoma* parasites); +Tryp = group of mosquitoes previously fed on a *Trypanosoma*-infected mouse. **: Combined p-value <0.01 (Fisher method) from the 3 independent biological replicates obtained for the infection prevalence (p = 0.006 for *P*. *yoelii*; p = 0.007 for *P*. *falciparum*) and for infection intensity (p = 0.001 for *P*. *yoelii* only). n = Total number of dissected mosquitoes. N = number of biological replicates.

Feeding mosquitoes with an initial *T*. *b*. *brucei* infectious blood meal significantly enhanced mosquito susceptibility to *P*. *yoelii* delivered in a second bloodmeal five days later, as compared to control mosquitoes that first fed on a naive mouse. The effect was significant for both infection prevalence (p = 0.006) and infection intensity (p = 0.001) (Fig 4A & 4B). When *P*. *falciparum* gametocytes were used for the second blood meal, infection prevalence but not intensity was increased in the mosquitoes exposed to trypanosomes in the first bloodmeal (p = 0.007) (Fig 4C).

**Fig 5:**
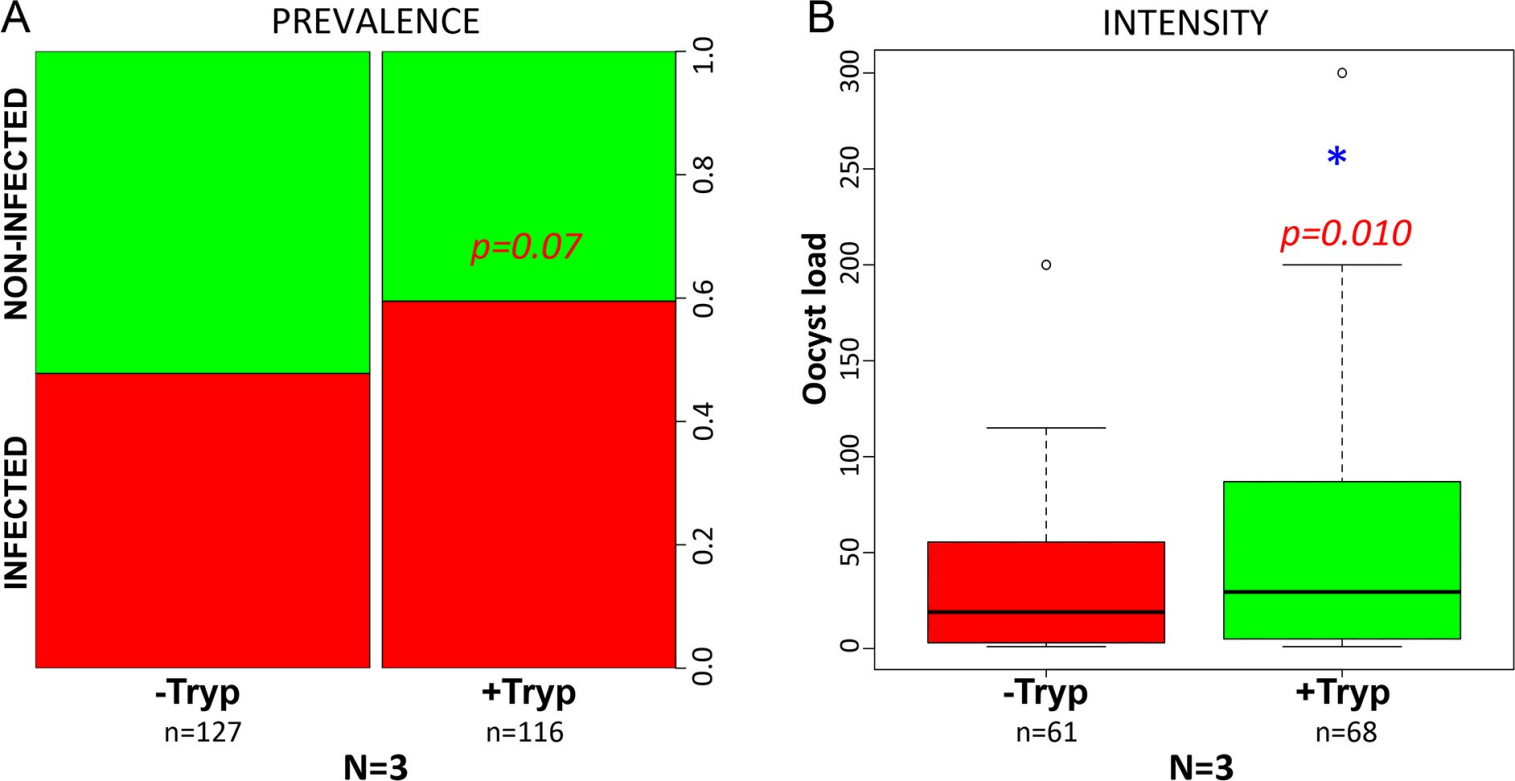
**The simultaneous presence of *T*. *b*. *brucei* and *P*. *yoelii* in *A*. *coluzzii* does not promote *Plasmodium* infection prevalence (A) but promotes infection intensity (B)**. In (A), red colour shows proportion of infected and green show proportion of uninfected individuals. -Tryp = group of mosquitoes fed on a *P*. *yoelii* infected mouse (but without *Trypanosoma* parasites); +Tryp = group of mosquitoes fed on a mouse co-infected with *T*. *b*. *brucei* and *P*. *yoelii* parasites. *: Combined p-value <0.05 (Fisher method) from the 3 independent biological replicates obtained for the infection intensity (p = 0.010). n = Total number of dissected mosquitoes. N = number of biological replicates.

Simultaneous exposure of mosquitoes to both *P*. *yoelii* and *T*. *b*. *brucei* also influenced the development of *Plasmodium*, similarly, although weakly, to successive exposure (infection prevalence, p = 0.07 and infection intensity, p = 0.010) (Fig 5).

In order to control for the possibility that potential immunomodulatory factors present in the bloodmeal from trypanosome-infected mice could be responsible for the increased mosquito susceptibility to *Plasmodium*, we fed mosquitoes on *in vitro* cultured *T*. *b*. *brucei* before challenging with *P*. *yoelii*. Sample sizes and statistical power were small because mosquitoes were weakly attracted by the medium; nevertheless, we observed a similar tendency of increased *P*. *yoelii* infection prevalence after exposure to trypanosomes (p-value = 0.07, S3A Fig). This result suggests that the agonistic effect of *T*. *b*. *brucei* ingestion on *A*. *coluzzii* vector competence was probably not due to mouse serum factors, but rather to the presence of trypanosomes in the mosquito midgut.

### Altered microbiome mediates the trypanosome enhancement of *Plasmodium* infection, but not *vitellogenin* decrease

An increase in midgut of Enterobacteriaceae has been positively correlated with *Anopheles* vector competence for *Plasmodium* [47]. We assessed by qPCR the level of Enterobacteriaceae in mosquitoes exposed to trypanosomes by targeting both the 16SrDNA and the 23SrDNA to increase the robustness of the measure. Consistent with the enhancement of *Plasmodium* infection phenotype, we found an increased abundance of Enterobacteriaceae as measured by both 16S and 23S in midguts of trypanosome-carrying mosquitoes (S4A Fig).

**Fig 6:**
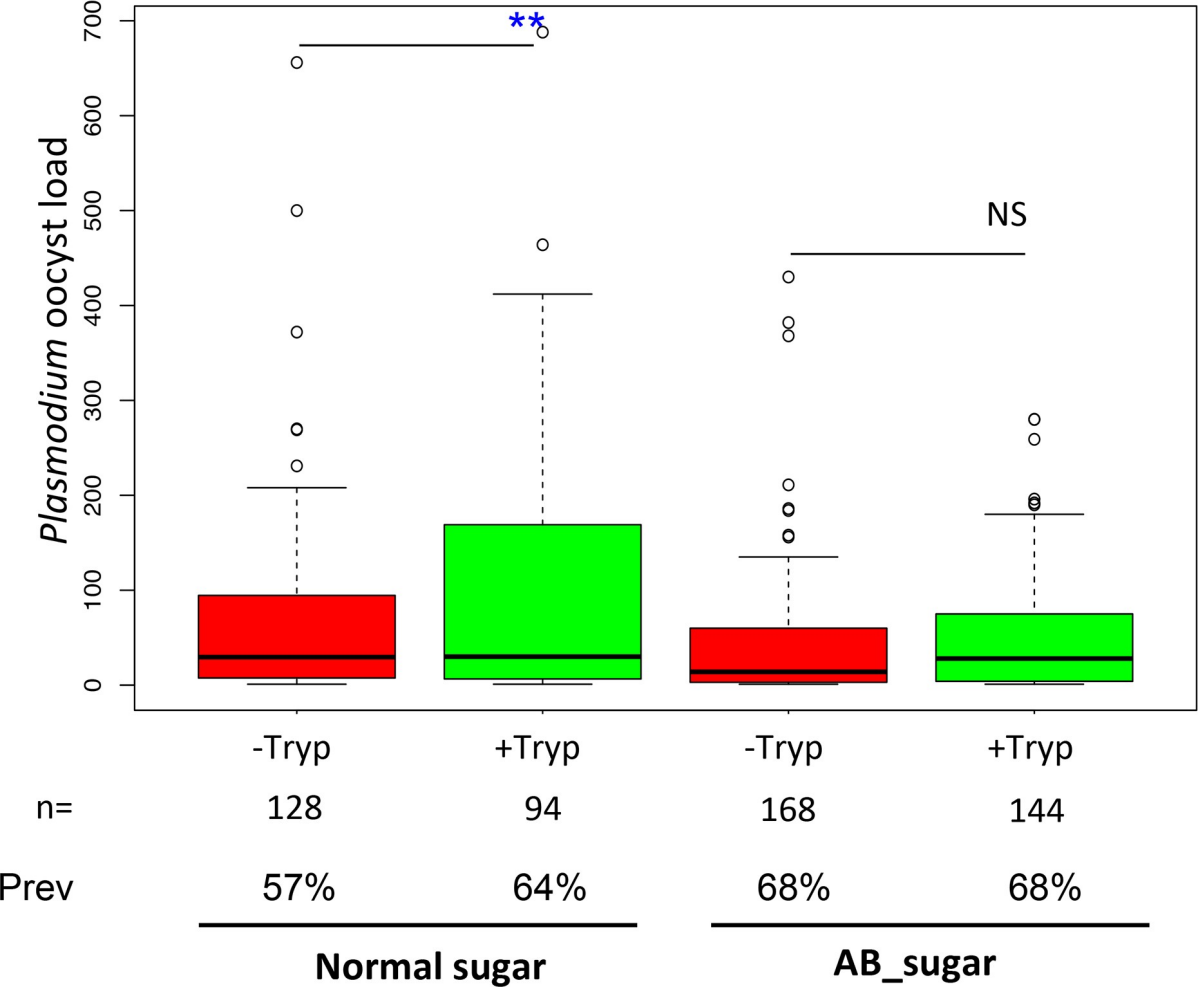
Increased susceptibility to *Plasmodium* in *Anopheles* mosquitoes exposed to *Trypanosoma* is microbiome-dependent. -Tryp = group of mosquitoes previously fed on a naive mouse (without *Trypanosoma* parasites); +Tryp = group of mosquitoes previously fed on a mouse infected with *Trypanosoma* parasites. Normal sugar = sugar without antibiotics; AB_sugar = sugar supplemented with antibiotics; **: Combined p-value <0.01 (Fisher method) from the 2 independent biological replicates obtained for the infection intensity (p = 0.007). NS: statistically not significant p-value (p = 0.201). n = Total number of dissected mosquitoes. Prev: infection prevalence in %.

As the relevant bacteria were antibiotic sensitive in treated mosquitoes (S4B Fig), we queried the effect of such treatment on the phenotype for *Plasmodium* susceptibility. Treatment of mosquitoes with antibiotics abolished the increased susceptibility of *Anopheles* to *Plasmodium* observed after trypanosome ingestion (Fig 6), indicating that the increased *Plasmodium* susceptibility was dependent upon the bacterial expansion.

**Fig 7:**
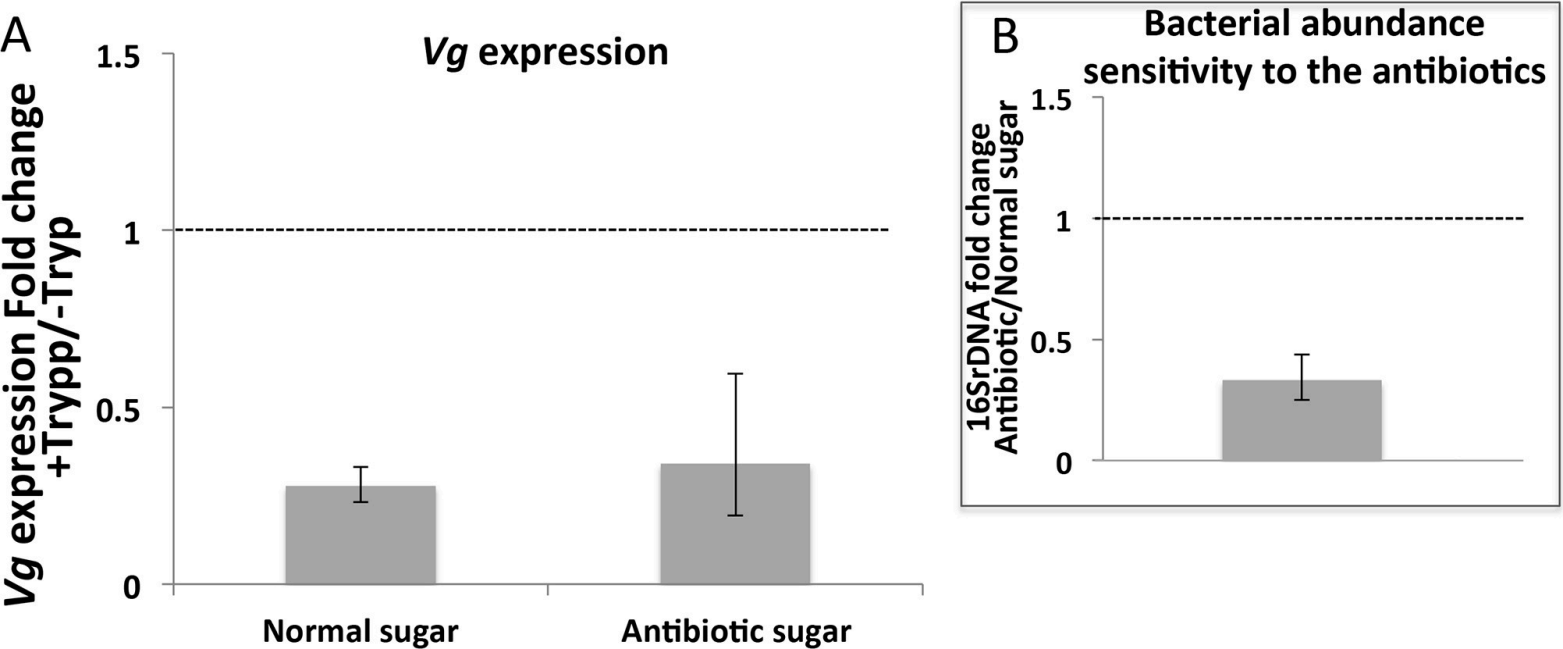
Decreased expression of *vitellogenin* in *T*. *b*. *brucei* infected background is not dependent on the bacterial abundance increase. (A) *Vitellogenin* (Vg) expression level was quantified by qPCR in “Normal sugar” and “Antibiotic sugar” backgrounds from mosquito samples fed on a naive mouse (-Tryp) or on a *Trypanosoma*-infected mouse (+Tryp). Each bar of the graph shows median fold change of *vitellogenin* expression in mosquitoes fed on mice infected by *Trypanosoma* (+Tryp) as compared to those fed on naive mice (doted line). The ribosomal protein *rps7* gene was used as an internal calibrator. The ratio of the normalized Vg expression in “+Tryp” *versus* “-Tryp” was calculated using triplicates from the same cDNA dilution. *: Statistically significant p-value (p<0.05) related to the deltaCt distribution between “+Tryp” and “-Tryp”. (B) Antibiotic efficiency on the bacterial abundance was verified by measuring the abundance of the bacteria (by qPCR detection of 16S rDNA) between “Antibiotic sugar” and “Normal sugar” (doted line) backgrounds 24h post-blood meal. The ratio of the normalized 16S rDNA detection in “Normal sugar” versus “Antibiotic sugar” backgrounds was computed using triplicates from the same cDNA dilution. In A & B, error bars show median absolute deviation computed by permutation from 3 independent biological experiments.

However, the observed decrease of *vitellogenin* expression in mosquitoes exposed to trypanosomes was not influenced by antibiotic treatment (Fig 7), indicating that the trypanosome effect on mosquito reproductive fitness was independent of the microbiome.

## Discussion

Human and animal trypanosome infections are often sympatric with malaria in Africa, and malaria vector mosquitoes can be exposed to both microbes in the same or successive bloodmeals. Although mosquitoes are not the natural hosts of the *T*. *brucei* cyclical development, we found that *T*. *b*. *brucei* parasites can transiently survive in the *A*. *coluzzii* midgut for at least 48h post-ingestion and differentiate into procyclic forms, before finally dying and being cleared by the mosquitoes. Strikingly, during this short period of time, the presence of trypanosomes alters the mosquito enteric microbiome, decreases the mosquito reproductive fitness via effects on female fertility, and enhances their susceptibility to *Plasmodium* infection. While the effect of trypanosomes ingestion on *Plasmodium* infection is microbiome dependent, the phenotype on their reproductive fitness is microbiome independent.

The vector microbiome is a major factor likely influencing parasite transmission through different mechanisms [3,47,50]. The presence of antibiotics in the mosquito midgut could increase its susceptibility to *Plasmodium* infection, highlighting beneficial effects of the microbiota on the mosquito resistance to *Plasmodium* infection [29,51]. In contrast, an imbalance in Enterobacteriaceae can promote *Anopheles* vector competence for *Plasmodium* [47]. Here, we show that the presence of live trypanosomes increases the abundance of midgut bacteria in *Anopheles*, which in turn increases vector competence for *Plasmodium*. In addition, we also demonstrated that Enterobacteriaceae, which were sensitive to the antibiotics treatment that abolished the *Plasmodium* susceptibility phenotype, were also elevated.

However, additional biological replicates might be necessary to increase the robustness of these findings related to the effect of trypanosome ingestion on the bacterial microbiome. In addition, some in-depth metagenomic analysis would be necessary to identify the specific bacterial taxa underlying the observed phenotypic effect. The mechanism leading to the gut bacterial expansion is not consistent with a resource competition between trypanosomes and the bacteria, as this would lead to an inverse phenotype. Two hypotheses could be proposed: i) trypanosomes could modify the gut environment in such a way that it would favour the development of some bacteria. ii) the immune factors controlling the gut homeostasis could be diverted towards the trypanosomes, which would lead to the gut bacteria expansion.

Bloodmeals containing trypanosomes reduce mosquito reproductive fitness, as measured both by *vitellogenin* expression and the egg hatching rate of the offspring. Although the increase in mosquito susceptibility to malaria parasite infection in the presence of trypanosomes was dependent on the midgut bacterial expansion, this was not the case for the decreased Vg expression. Reduction of host fecundity has been reported in other parasitized insects [52]. However, it is not clear whether host fecundity reduction reflects parasite manipulation of the vector in order to increase contacts with new hosts and promote its own transmission or nutrient competition with the insect host, or both [53–55]. The induction of the *Vitellogenin* and *Lipophorin* through the 20E pathway is triggered by amino acids and nutrients made during the blood digestion process [42,49,44]. The presence of active trypanosomes in the midgut during at least the first 48h following infection could act as a host competitor, and therefore reduce the amount of nutrients available for *Vg* and *Lp* induction through the 20E pathway. In other words, *Trypanosoma* survival in the mosquito midgut might alter the activation of the 20E pathway and thus the expression of downstream factors such as *Vg* and *Lp*, which in turn would impact on the reproductive fitness. Nevertheless, although an alteration of the 20E pathway has been previously correlated with a decreased susceptibility to *Plasmodium* [44], we found that the presence of trypanosomes induces a decrease of *Vg* and *Lp* expressions but an increase of the *Anophele*s vector competence for *Plasmodium*. Therefore, the effect of trypanosomes on *Anopheles* vector competence would be mostly related to their impact on the microbiome rather than on the 20E pathway. In *Glossina*, the activation of immune responses by immunogenic wild type trypanosome strains reduces reproductive output as well as the milk gland protein levels (required for larval development), while infections with non-immunogenic trypanosomes do not [56], revealing a balanced interplay between immune activity and reproduction in tsetse flies. In *Anopheles*, few studies have highlighted a potential trade-off between immunity and reproduction. In the mosquitoes *Anopheles stephensi* and *Anopheles gambiae*, *Plasmodium yoelii nigeriensis* was shown to influence the balance between reproductive fitness and immune defences, which was linked to an immune pathway inducing apoptosis in ovarian follicle cells [57,58]. Furthermore, in *A*. *gambiae*, the low *Plasmodium* survival phenotype associated with the *Lp*/*Vg* knockdowns relies on an anti-plasmodial TEP1-related mechanism [49]. Considering our current data, it is tempting to postulate that *T*. *b*. *brucei* could modulate the interplay between immunity and reproduction in *A*. *coluzzii*. However, our results showed that *T*. *b*. *brucei* only weakly modulated immune factors and that the increased susceptibility to *Plasmodium* was rather dependent on the gut bacterial flora than on a direct effect of trypanosome cells. However, in contrast to the vector competence phenotype, the effects on the mosquito reproductive fitness triggered by *T*. *b*. *brucei* were not dependent on the gut microbiota. Hence these two phenotypes are likely the result of distinct, as yet undescribed, mechanisms.

Given the sympatric distribution of malaria and trypanosomiases, it would be interesting to assess whether these experimentally trypanosome-induced effects on laboratory mosquito strains could also occur in nature, with some potential consequences for the epidemiology of malaria. Although *T*. *brucei* is a common parasite and sympatric with *P*. *falciparum* in a number of African regions, mosquitoes do not successfully transmit it; nevertheless, they could be exposed to it. More information would be necessary to determine the complex relationships between multiple parasite infections and interpret transmission dynamics in nature. Other less studied protists that are not necessarily human pathogens but rather insect parasites, such as the monoxenic parasite *Crithidia fasciculata* for instance, are probably as abundant in nature, and may also affect mosquito biology in a similar manner. In total, adult *Anopheles* female mosquitoes are likely to be exposed to a large panel of microorganisms, bacteria and viruses that would alter their vector competence in distinct ways [1]. Overall, our results emphasize the potential influence of eukaryotic organisms in combination with the bacterial microbiome on *Anopheles* vector competence for *Plasmodium*.

## Supporting information

S1 Fig *APL1B* overexpression 48h post-trypanosome ingestion depends on the midgut bacterial expansion. (A) Relative quantification of *APL1B* and *GNBPB1* gene expression in females fed on trypanosome-infected mouse (+Tryp) or on naive mouse (-Tryp) and maintained on sucrose, using expression of the ribosomal protein *rps7* gene as an internal calibrator. The dotted line represents the median expression in the control (-Tryp). (B) Relative quantification of *APL1B* gene expression in females fed on trypanosome-infected mouse (+Tryp) or on naive mouse (-Tryp) and maintained on sucrose supplemented with an antibiotic cocktail, using expression of the ribosomal protein *rps7* gene as the internal calibrator. In A and B, the ratio of the normalized gene of interest in (+Tryp) *versus* (-Tryp) control was computed using triplicates from the same cDNA dilution. Error bars show median absolute deviation computed by permutation from 3 technical replicates for each independent biological replicate (EXP1 and EXP2).
(TIF)

S2 Fig Trypanosome parasites displayed a direct effect on *Anopheles* reproductive fitness. A & B. Relative quantification of *Lp* and *Vg* gene expression in females fed on trypanosome-containing blood (+Tryp) or on blood without trypanosomes (-Tryp), using expression of the ribosomal protein *rps7* gene as the internal calibrator. The dotted line represents the median expression in the “-Tryp” control group. The graph in A shows qPCR results from the mouse infection system, while the graph in B shows qPCR from cultured trypanosomes mixed with sheep blood. *: Statistically significant p-value (p<0.05) related to the deltaCt distribution between “+Tryp” and “-Tryp” across 3 independent biological replicates. C. The graph shows the number of laid eggs per individual females fed on cultured trypanosomes mixed with sheep blood. The differences between the two groups of females (+Tryp) *versus* (-Tryp) was analysed using a Wilcoxon signed-rank non-parametric test; n = number of individual females from each group.
(TIF)

S3 Fig Ingestion of cultured *T. b. brucei* increases the infection prevalence of *A. coluzzii* to *P. yoelii*. Panel A shows results of infection prevalence. Red colour shows proportion of infected and green shows proportion of uninfected individuals. Panel B shows result of infection intensity. -Tryp = group of mosquitoes previously fed with culture medium only (without *Trypanosoma* parasites); +Tryp = Group of mosquitoes fed with culture medium containing *T*. *b*. *brucei*. N = number of biological replicates. Combined p-value was done using Fisher method from the 3 independent biological replicates. n = Total number of dissected mosquitoes.
(TIF)

S4 Fig *Trypanosoma* ingestion increases the abundance of Enterobacteriaceae family in *A. coluzzii* at day 5 post-feeding. (A) 16S and 23S rDNA detection of Enterobacteriaceae was performed by qPCR at day 5 (D5) post-blood meal using the expression of the ribosomal protein *rps7* gene as the internal calibrator. The two couple of primers (16S and 23S were both used to increase the robustness of the results. The graph shows median fold change of the Enterobacteriaceae load in midguts of mosquitoes challenged with trypanosome as compared to mosquito fed on naive mouse (doted line). With Tryp = group of mosquitoes previously fed on a *Trypanosoma*-infected mouse. The ratio of the normalized 16S (or 23S) rDNA detection in “With Tryp” *versus* “Naive” was computed using triplicates from the same cDNA dilution. Error bars show median absolute deviation computed by permutation from 3 experiments. *: Statistically significant p-value (p<0.05) related to the deltaCt distribution between “+Tryp” and “Naive”. NS: Non-significant p-value. (B) Antibiotic efficiency on Enterobacteriaceae family. 16S and 23S rDNA detection of Enterobateriaceae was performed by qPCR at day 5 (D5) post-naive blood meal using sample from mosquitoes treated or not with antibiotics. Expression of the ribosomal protein *rps7* gene was used as the internal calibrator. The dotted line represents the level of 16S and 23S rDNA in the normal sugar background (without antibiotic). The ratio of the normalized 16S (or 23S) rDNA detection in “AB sugar” (with antibiotic) *versus* “Normal sugar” (without antibiotics) was computed using triplicates from the same cDNA dilution. Error bars show median absolute deviation computed by permutation from 3 independent biological experiments.
(TIF)

S1 Movie Procyclic-like trypanosomes swimming out of a mosquito midgut 48h post-ingestion. *Anopheles* midguts were dissected in PBS 48h post-trypanosome ingestion and scrutinized under a microscope at the 100x magnification. Trypanosomes with a procyclic trypomastigote shape were found to be highly motile in all midguts.
(MPEG)

S1 Table RNAseq identification of 13 genes, which expressions are modulated 48h after *T. b. brucei* ingestion. Among these 13 genes, two are immune–like genes: *3-Glucan binding protein* and *APL1B* (highlighted in pink). Results come from three independent biological experiments. For each gene, differential expressions between *T*. *b*. *brucei*-infected and naive feeding backgrounds are expressed in Log2 fold change, p-values and adjusted p-values (padj) to highlight statistical difference.
(XLSX)

S2 Table RNAseq analysis at 24h after *T. b. brucei* ingestion. For each gene, differential expressions between *T*. *b*. *brucei*-infected and naive feeding backgrounds are expressed in Log2 fold change, p-values and adjusted p-values (padj) to highlight statistical difference. The count values are noted for each gene and for each three biological replicates: NaiveMouse_1 = mosquito fed on naive mouse for the first biological replicate; TrypaMouse_1 = mosquito fed on *T*. *b*. *brucei*-infected mouse for the first biological replicate.
(XLS)

S3 Table RNAseq analysis at 48h after *T. b. brucei* ingestion. For each gene, differential expressions between *T*. *b*. *brucei*-infected and naive feeding backgrounds are expressed in Log2 fold change, p-values and adjusted p-values (padj) to highlight statistical difference. The count values are noted for each gene and for each three biological replicates: NaiveMouse_1 = mosquito fed on naive mouse for the first biological replicate; TrypaMouse_1 = mosquito fed on *T*. *b*. *brucei*-infected mouse for the first biological replicate.
(XLS)
