# Supplementary figures and images for: Exposure of Anopheles mosquitoes to trypanosomes reduces reproductive fitness and enhances susceptibility to Plasmodium
Source: PLoS Negl Trop Dis. 2020 Feb 7;14(2):e0008059. doi: 10.1371/journal.pntd.0008059 (PMC7032731; doi:10.1371/journal.pntd.0008059)

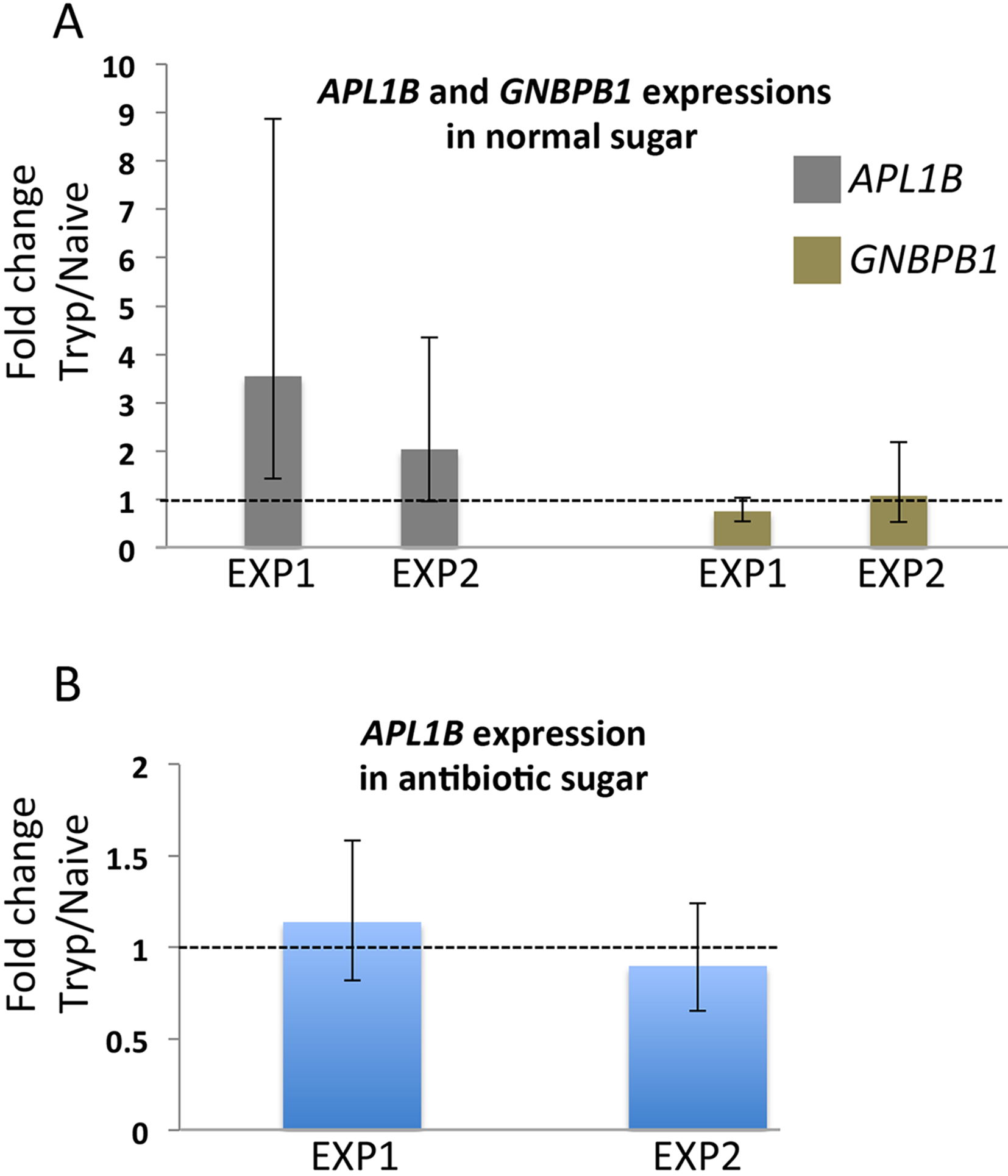

Supplement: S1 Fig — (A) Relative quantification of APL1B and GNBPB1 gene expression in females fed on trypanosome-infected mouse (+Tryp) or on naive mouse (-Tryp) and maintained on sucrose, using expression of the ribosomal protein rps7 gene as an internal calibrator. The dotted line represents the median expression in the control (-Tryp). (B) Relative quantification of APL1B gene expression in females fed on trypanosome-infected mouse (+Tryp) or on naive mouse (-Tryp) and maintained on sucrose supplemented with an antibiotic cocktail, using expression of the ribosomal protein rps7 gene as the internal calibrator. In A and B, the ratio of the normalized gene of interest in (+Tryp) versus (-Tryp) control was computed using triplicates from the same cDNA dilution. Error bars show median absolute deviation computed by permutation from 3 technical replicates for each independent biological replicate (EXP1 and EXP2). (TIF) [file pntd.0008059.s001.tif]

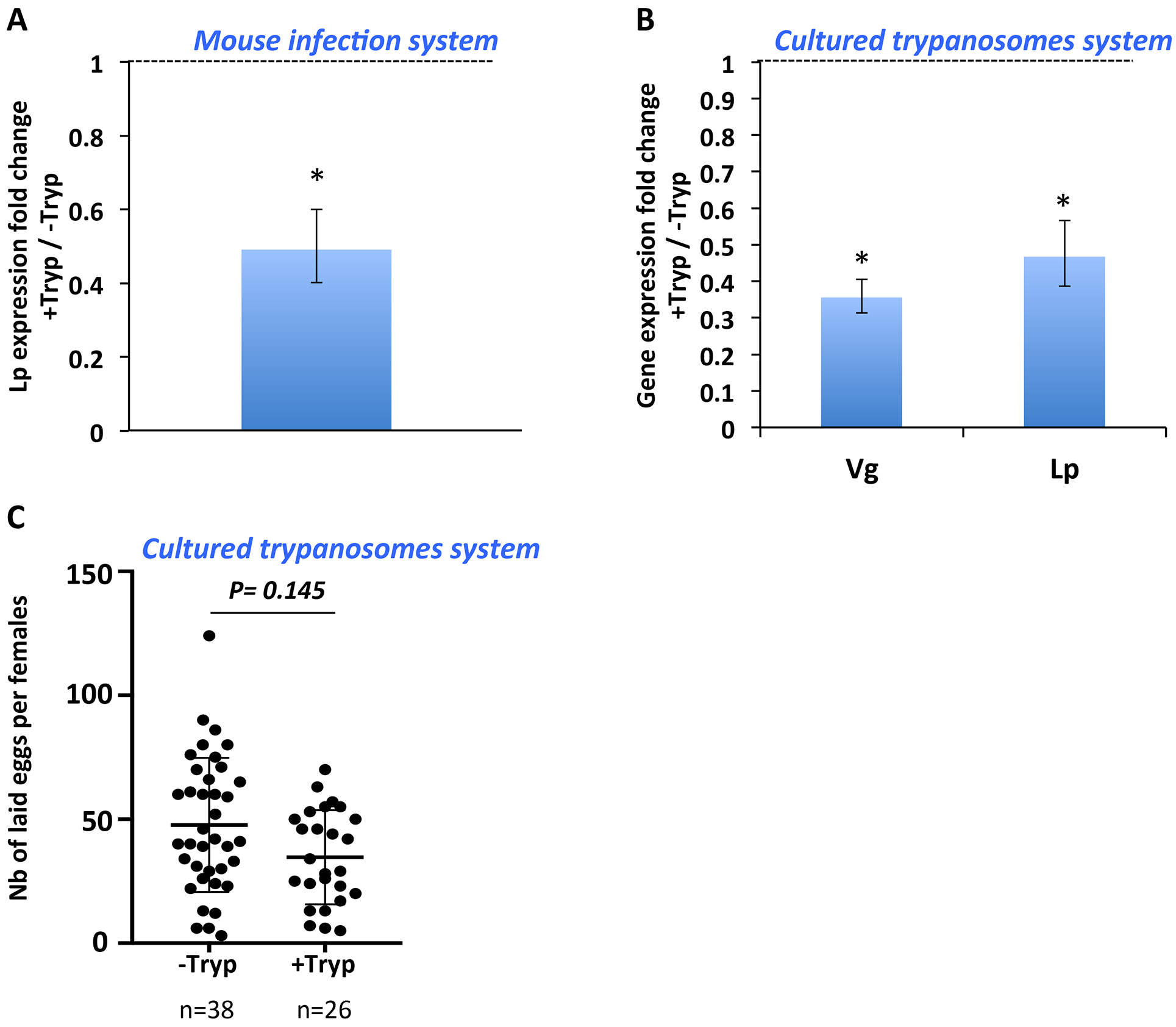

Supplement: S2 Fig — A & B. Relative quantification of Lp and Vg gene expression in females fed on trypanosome-containing blood (+Tryp) or on blood without trypanosomes (-Tryp), using expression of the ribosomal protein rps7 gene as the internal calibrator. The dotted line represents the median expression in the “-Tryp” control group. The graph in A shows qPCR results from the mouse infection system, while the graph in B shows qPCR from cultured trypanosomes mixed with sheep blood. *: Statistically significant p-value (p<0.05) related to the deltaCt distribution between “+Tryp” and “-Tryp” across 3 independent biological replicates. C. The graph shows the number of laid eggs per individual females fed on cultured trypanosomes mixed with sheep blood. The differences between the two groups of females (+Tryp) versus (-Tryp) was analysed using a Wilcoxon signed-rank non-parametric test; n = number of individual females from each group. (TIF) [file pntd.0008059.s002.tif]

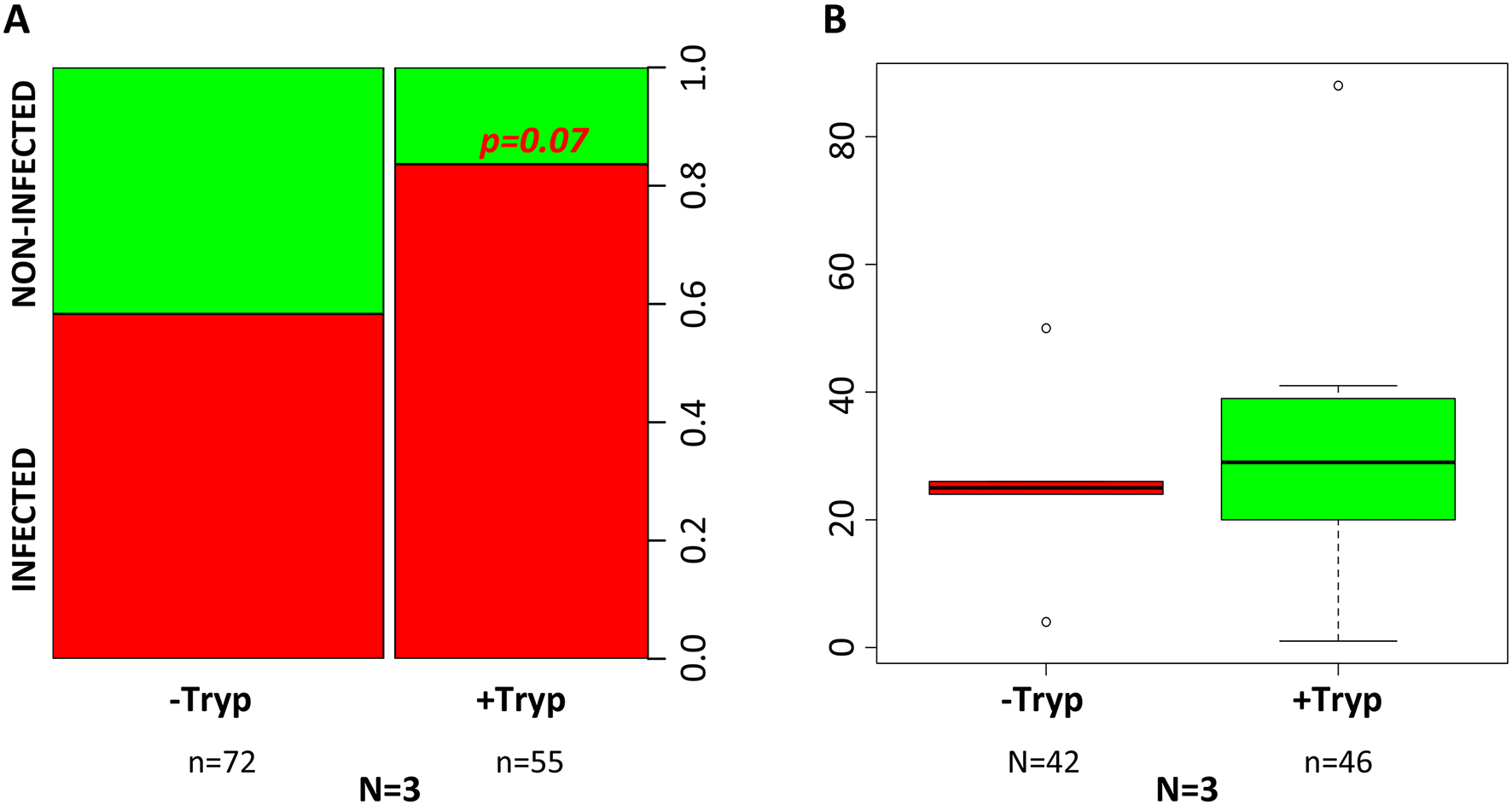

Supplement: S3 Fig — Panel A shows results of infection prevalence. Red colour shows proportion of infected and green shows proportion of uninfected individuals. Panel B shows result of infection intensity. -Tryp = group of mosquitoes previously fed with culture medium only (without Trypanosoma parasites); +Tryp = Group of mosquitoes fed with culture medium containing T. b. brucei. N = number of biological replicates. Combined p-value was done using Fisher method from the 3 independent biological replicates. n = Total number of dissected mosquitoes. (TIF) [file pntd.0008059.s003.tif]

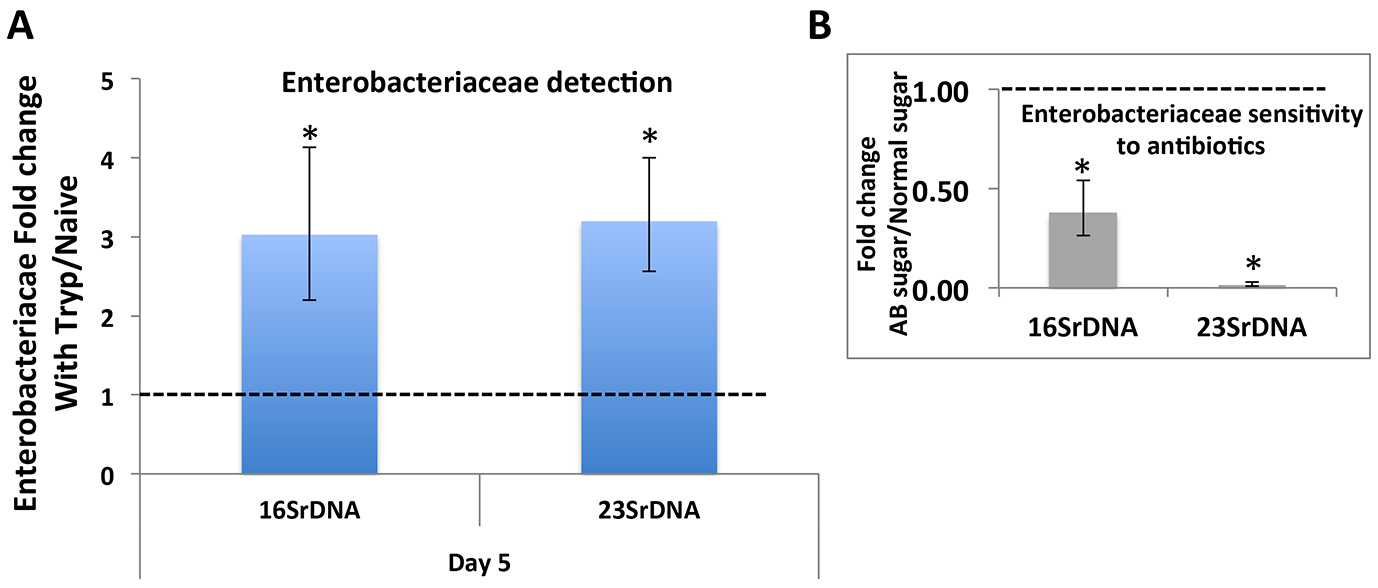

Supplement: S4 Fig — (A) 16S and 23S rDNA detection of Enterobacteriaceae was performed by qPCR at day 5 (D5) post-blood meal using the expression of the ribosomal protein rps7 gene as the internal calibrator. The two couple of primers (16S and 23S were both used to increase the robustness of the results. The graph shows median fold change of the Enterobacteriaceae load in midguts of mosquitoes challenged with trypanosome as compared to mosquito fed on naive mouse (doted line). With Tryp = group of mosquitoes previously fed on a Trypanosoma-infected mouse. The ratio of the normalized 16S (or 23S) rDNA detection in “With Tryp” versus “Naive” was computed using triplicates from the same cDNA dilution. Error bars show median absolute deviation computed by permutation from 3 experiments. *: Statistically significant p-value (p<0.05) related to the deltaCt distribution between “+Tryp” and “Naive”. NS: Non-significant p-value. (B) Antibiotic efficiency on Enterobacteriaceae family. 16S and 23S rDNA detection of Enterobateriaceae was performed by qPCR at day 5 (D5) post-naive blood meal using sample from mosquitoes treated or not with antibiotics. Expression of the ribosomal protein rps7 gene was used as the internal calibrator. The dotted line represents the level of 16S and 23S rDNA in the normal sugar background (without antibiotic). The ratio of the normalized 16S (or 23S) rDNA detection in “AB sugar” (with antibiotic) versus “Normal sugar” (without antibiotics) was computed using triplicates from the same cDNA dilution. Error bars show median absolute deviation computed by permutation from 3 independent biological experiments. (TIF) [file pntd.0008059.s004.tif]
